# Nanofibers and Nanotextured Materials: Design Insights, Bactericidal Mechanisms and Environmental Advances

**DOI:** 10.3390/nano13212891

**Published:** 2023-10-31

**Authors:** Touseef Amna, M. Shamshi Hassan

**Affiliations:** 1 Department of Biology, College of Science, Al-Baha University, Albaha 65799, Saudi Arabia; 2 Department of Chemistry, College of Science, Al-Baha University, Albaha 65799, Saudi Arabia

**Keywords:** antimicrobial, nanofibers, electrospinning, bactericidal mechanisms, environmental remediation

## Abstract

Antibiotic resistance is rising and poses a serious threat to human health on a worldwide scale. It can make it more difficult to cure common infections, raise medical expenditures, and increase mortality. In order to combat the development of biofilms and treat fatal bacterial infections, multifunctional polymeric nanofibers or nanotextured materials with specific structural features and special physiochemical capabilities have become a crucial tool. Due to the increased antibiotic resistance of many diseases, nanofibers with antibacterial activity are essential. Electrospinning is a flexible process able to produce fine fibers with specified properties by modifying variables such as the concentration of the solution, the feed flow, and the electric voltage. Substantial advancements have been made regarding the formation of nanofibers or nanotextured materials for a variety of applications, along with the development of electrospinning techniques in recent years. Using well-defined antimicrobial nanoparticles, encapsulating traditional therapeutic agents, plant-based bioactive agents, and pure compounds in polymer nanofibers has resulted in outstanding antimicrobial activity and has aided in curing deadly microbial infections. A plethora of studies have revealed that electrospinning is an effective technique for the production of antimicrobial fibers for the environmental, biomedical, pharmaceutical, and food sectors. Nevertheless, numerous studies have also demonstrated that the surface characteristics of substrates, such as holes, fibers, and ridges at the nanoscale, have an impact on cell proliferation, adhesion, and orientation.

## 1. Introduction

Nanoparticles and nanostructured materials represent a dynamic field of research, owing to their influence across a wide range of application domains. Significant advancements have been made due to recent breakthroughs in the preparation of nanostructured materials and in vital knowledge regarding their characteristics. Bacterial antibiotic resistance, and the rise of multidrug-resistant (MDR) microbes or bugs, have created a universal problem in recent decades. The use of inadequate antibiotics to treat MDR bacteria has resulted in the development of unusual resistances that have astonishingly propagated from corner to corner via the atmosphere, individuals, and faunae. 

Materials with a nanofibrous morphology have the potential to be employed to address a variety of environmental problems, including wastewater treatment, water disinfection, and air purification. In many biological systems, fascinating hierarchical structures frequently develop. Various hierarchical structures, including zero-dimensional particles, one-dimensional nanofibers, two-dimensional substrates, and even bulks, are now exploited in biomedicine. These include nanofibers that can create 1–3 dimensional substrates, and have a variety of sizes (nanometers or micrometers) and rich morphologies (for instance, rods, wires or tubes). For the purpose of creating nanofibers, a number of techniques, including electrospinning, chemical vapor deposition, melt spinning, solution spinning, and sintering, have been developed. A quick and affordable technique able to create micronanometer continuous nanofibers using a variety of polymers or compounds is electrospinning technology. The generated electrospun nanofibers have a high porosity, large specific surface area, and compact size, making them useful in a variety of biological applications, such tissue engineering, regenerative medicine, and wound dressings. An overview of the electrospun nanofibers used in antibacterial materials, tissue engineering and antimicrobial wound dressings is presented here. Additionally, new advances in the production of antimicrobial nanofibers utilized for aforementioned purposes are scrupulously described [1]. Furthermore, the utilization of polymeric nanofibers loaded with antibiotics to kill bacteria is garnering attention to a greater extent. In particular, mainstream research has examined the intrinsic structure, adjustable structure, elements, and features of nanofibers, which enable the construction of drug-laden nanofibers with a persistent discharge configuration for application in drug transport [2,3,4]. Biocide material is often incorporated into fibers during the manufacture of antimicrobial nanofibers or nanotextured materials. This can be accomplished in a number of ways, including uniformly mixing the bioactive molecule into the polymer solution preceding electrospinning, encasing it in a fiber core via coaxial electrospinning, encasing it in nanostructures before diffusing it in electrospinning solution, or assigning it to the fiber’s surface. To date, antibiotics, biocides, metallic nanoparticles, metal oxide nanoparticles, and organic chemicals have all been employed as active ingredients [5]. 

Electrospun nanofiber scaffolds have recently been shown to be excellent nanoscale therapeutic devices, owing to their physicochemical characteristics; these can be adjusted and made suitable for a variety of applications requiring antibacterial qualities. The design requirements and characteristics of electrospun nanofibers that can be used to enhance their therapeutic activities are briefly highlighted herein. Nanofibrous structures have several inherent qualities that characterize their use as functional materials, particularly for antibacterial or antimicrobial purposes. For instance, a fiber’s structure should be able to biomimic the extracellular matrix (ECM) of tissue at the nanometric scale, fashioning a favorable atmosphere for the regeneration of the target region and enabling healing processes. They can affect cell migration, linkage, distinction, and renewal, in addition to having topographical fibers that resemble the original ECM architecture. In addition, the porosity of nanofibers, which permits high-surface and wetting absorptivity and, consequently, affects cell proliferation, vascularization, and mechanical firmness, has a significant impact on their performance. Furthermore, the fractal structure of nanofibers, with its linked nanopores, superior surface energy, reactivity, and elevated thermal and electric conductivities, might deter the development of cells and inhibit microbial penetration. Electrospun nanofibers have the potential to be effective as antimicrobial materials for all the aforementioned reasons [6].

Microorganisms are naturally able to modify their structural features and structures to lessen the effectiveness of antibiotics. By impairing bacterial walls and snooping with their DNA, RNA, or vital proteins, antibiotics kill bacteria. However, by altering their gene and protein expressions, bacterial cells may also acclimatize to environmental provocations. As more strains of bacteria develop multidrug resistance, antibiotic resistance in bacteria will rise. Due to this phenomena, it is urgent that non-antibiotic alternatives to antibiotics, to be employed as alternate antimicrobial therapies for these very resistant microorganisms, are found. Nanostructured materials are attracting interest and recognition in biomedical usage as a result of the development of nanotechnology. In particular, electrospun polymeric nanofibers have distinctive physical and chemical characteristics, such as specific dimensions, silhouettes, and apparent chemistries, that affect their healing action. As a result, they are flexible enough to be readily customized for antimicrobial treatment [7,8,9,10,11].

The aim and purpose of this Special Issue, entitled “Prospects of Bioinspired and Biomimetic Materials”, was to summarize the current state of ongoing research in the area of nanostructured and nanofibrous materials, with a distinctive focus on their antibacterial [12], bactericidal or bacteriostatic mechanisms [7,9,13], biomedical applications [14,15,16,17,18], and environmental advances [19,20,21,22,23,24,25]. Original research pieces illustrating and enumerating key developments in the aforementioned study domains were accepted for submission to this Special Issue. This Special Issue then assembled nine carefully chosen original research papers addressing a variety of subjects related to nanostructured materials, which were well characterized, ranging from basic study to advanced practical applications. Nevertheless, the key objectives of the contributed research investigations were based on antibacterial mechanisms, biomedical applications, and environmental remediation. The accomplishment of this Special Issue was made possible by the research efforts and skills of renowned scientists from diverse universities and research institutions.

Here is a summary of the contributions submitted to this Special Issue. 

In one study, a successful electrospinning approach was employed to construct CeO_2_ and SnO_2_ composite nanofibers. An analysis using scanning and transmission electron microscopy revealed that CeO_2_ and SnO_2_ nanofibers have an average diameter of 170 nm. The CeO_2_–SnO_2_ composite performed more efficiently when the methylene blue dye was subjected to photocatalytic degradation. *Escherichia coli* was also used as a representative bacterium to screen the aforementioned nanofibers for antibacterial activity, and the CeO_2_ and SnO_2_ composite nanofibers exhibited outstanding activity. The results of this research offer new opportunities for the purification of chemical and biological pollutants in water and the use of electrode materials in energy storage devices, which would significantly aid environmental remediation procedures [22].

Several deadly illnesses in humans and animals are induced by environmental pollution, particularly water contamination from dyes, heavy metal ions, and biological infections. The materials and techniques required for water purification are costly. As a result, there is an urgent need for low-cost materials for wastewater treatment. Considering this, butchered cow bone debris from the Najran area of Saudi Arabia were gathered and subjected to a thermal-based synthesis method for the preparation of hydroxyapatite. A composite composed of manganese ferrite and hydroxyapatite was then synthesized. The nanocomposite was classified using a variety of complex techniques, including X-ray diffraction (XRD), field emission scanning electron microscopy (FE-SEM), energy-dispersive X-ray (EDX), transmission electron microscopy (TEM), UV–Vis, photoluminescence (PL), and Fourier transform infrared (FT-IR). This composite was found to have an incomparable photodegradation efficiency for industrial waste pollutants. Additionally, this composite demonstrated outstanding bacteriostatic activity towards the microorganisms *E. coli* and *S. aureus*, which cause acute waterborne illnesses. The results of this investigation revealed that, compared to virgin hydroxyapatite, the integration of manganese ferrite greatly enhanced both antibacterial and photocatalytic effects [21].

Similarly, the main aim of this study was to create a unique heterostructure from camel waste bones using an affordable and environmentally friendly approach in order to purify various waste liquids. Camel bones were used to create hydroxyapatite by employing a hydrothermal process. The produced hydroxyapatite was used to create a heterostructure of cerium oxide–hydroxyapatite functionalized with chitosan, a biopolymer. Because chitosan is a plentiful natural polysaccharide, it has several impressive qualities that make it an excellent adsorbent for eliminating colorants and other unwanted molecules from water. These qualities include accessibility, affordability, hydrophilicity, biocompatibility, and biodegradability. The remarkable bacteriostatic capacity of this heterostructure regarding *E. coli,* which is responsible for major waterborne illnesses, was another noteworthy feature. It was interesting that adding cerium oxide and chitosan to hydroxyapatite significantly enhanced its antibacterial and adsorption characteristics over that of pristine hydroxyapatite. Additionally, a unique heterostructure developed out of recycled superfluous camel bones that helps to reduce water pollution has mostly been engendered by the dye industry [24].

In another study, the effectiveness of micro- and nano-clay as a cheap substance for removing crystal violet (CV) dye from an aqueous solution was investigated. Thermodynamic observations demonstrated the exothermic, spontaneous nature of CV dye adsorption on both micro- and nano-clays. Because of this, the natural micro- and nano-clays were used potent and promising adsorbents for the elimination of CV dye from an aqueous solution [26].

Apart from environmental and water pollution, cancer and microbial infections pose great risks to health and are a prevalent cause of mortality globally. A key component of the drug discovery process is the synthesis of medicinal molecules from natural and indigenous resources. As a result, the anticancer and antibacterial properties of extracts and fractions of *Dodonaea viscosa* and *Juniperus procera* were examined in one study. Two fractions were discovered to have potential anticancer and antibacterial properties. Further, modifications were made to active fractions in order to generate ZnO composites, and these were examined using SEM, XRD, TGA, and EDX. Furthermore, these nanocomposites were revealed to be safe when evaluated for cytotoxicity against typical fibroblasts. The active fractions were also subjected to GC-MS analysis to identify potential phytochemicals that may be accountable for the aforementioned activities [27].

Similarly, in another research study, the objective of the work was to evaluate the biocompatibility of polyurethane (PU) nanocomposites grafted with polyhedral oligomeric silsesquioxane (POSS) and their potential application as materials for muscle tissue regeneration. POSS nanoparticles exhibit effective nucleation and significantly enhance the mechanical, thermal, and biocompatibility of the resulting composites. This study examined the antibacterial capability, anchoring, propagation, communication, and differentiation of C2C12 on PU and POSS-grafted PU. As a result of the higher free energy of the POSS molecules and their anti-inflammatory potential, the abovementioned nanocomposites exhibited improved cell adherence. Because these nanofibers were found to be safe, biomimetic scaffolds offer great potential regarding cellular research and muscle regeneration [28].

Interestingly, a green redox chemistry procedure was used to create gold (Au), silver (Ag), and palladium (Pd) nanoparticles by reducing the metal salt precursor with glucose, while also utilizing polyvinylpyrrolidone (PVP) as a stabilizing and capping agent. For the cytoskeleton, actin and mitochondria, respectively, the quantitative analysis of Mitotracker Deep Red and Actin Green exhibits a tendency for disturbance that is consistent with the results of the wound scratch experiment. With regard to the untreated control cells, the Pd NP-treated cells showed the greatest change, followed by Au NP-treated cells; meanwhile, the Ag NP-treated cells showed the least disruptive change, despite being statistically significantly different from the control in the case of mitochondria [29].

Furthermore, in another investigation, a variety of Retinin-like proteins and techniques for their metallization, employing nickel, silver, and copper ions to enhance the adaptability of the bionic nanocoatings, were applied. The optimum methods for creating secure and anti-infective metalized bionic nanocoatings were identified via the comparative analysis of the ensuing bactericidal, antiviral, and cytotoxic capabilities. The more extensive application of these standards to various public surfaces may represent a secure and affordable strategy via which to minimize the risk of dangerous illnesses [30].

Equally, for the same reason, two distinct nanotechnological methods were employed to create water-soluble preparations of pyrazole derivative 3-(4-chlorophenyl)-5-(4-nitrophenylamino)-1H-pyrazole-4-carbonitrile (CR232), which was revealed to exert an in vitro antiproliferative effect on several cancer cell types. Most significantly, CR232 was first trapped in a biodegradable fifth-generation dendrimer encompassing lysine (G5K), which is significant since this was performed without utilizing hazardous chemical solvents or additives that might be dangerous to humans [31].

In summary, the studies included in this Special Issue demonstrate both the existing and flourishing thoughtfulness regarding the design, preparation, characterization, and possible applications of functional nanofibers or nano-textured materials. These significant contributions to fundamental research will open up diverse avenues for growth and innovation, enhancing the application of these nanotextured materials and fibers in technological fields.
